# Mechanisms and durability of residential treatment for co-occurring gambling and substance use disorders: a mixed-methods eight-year follow-up study

**DOI:** 10.3389/fpsyt.2026.1835519

**Published:** 2026-07-06

**Authors:** Jonah Im, Sylvia Silver, Fiona Donovan, Aiden Wong, Emma Le, Akul Goel, Ajay Tunikipati, Brad Ruderman, Timothy Fong

**Affiliations:** 1 David Geffen School of Medicine, University of California, Los Angeles, Los Angeles, CA, United States; 2 College of Letters and Science, University of California, Los Angeles, Los Angeles, CA, United States; 3 Gambling Studies Program, University of California Los Angeles (UCLA), Los Angeles, Los Angeles, CA, United States

**Keywords:** gambling disorder, residential treatment, long-term outcomes, mixed methods, recovery, relapse, recovery trajectories, re-abstinence

## Abstract

**Introduction:**

Gambling disorder is associated with substantial psychosocial harm, yet long-term outcome data for residential treatment programs remain limited.

**Methods:**

This mixed-methods study examined outcomes among 36 former residential treatment clients assessed between six months and eight years following discharge from a program treating co-occurring gambling and substance use disorders. Structured interviews evaluated gambling behavior, psychiatric symptoms, substance use, and functional outcomes, supplemented by qualitative thematic analysis.

**Results:**

Sixty-one percent reported gambling following discharge; however, participants demonstrated significant improvements in gambling craving strength, life satisfaction, gambling-related interference, and alcohol use. Employment increased from 25.0% to 66.7%. Treatment non-completion was associated with earlier return to gambling and higher odds of post-discharge gambling. Qualitative analysis identified environmental restructuring, urge-management skill development, structured routines, and post-discharge support as key recovery mechanisms.

**Discussion:**

Residential treatment may support durable improvements in functioning and gambling-related distress among individuals with severe gambling disorder, although causal conclusions are limited by the observational design.

## Introduction

Gambling disorder is a behavioral addiction associated with substantial financial, psychological, and social harm. Large epidemiologic studies identified lifetime prevalence of problem/pathological gambling in adult populations to be 1.29% and moderate risk/at-risk gambling prevalence to be 2.43% globally, with higher rates when subclinical gambling-related problems are included (1, 2). Psychiatric comorbidity is highly prevalent, with up to 96% of individuals in some studies meeting criteria for at least one co-occurring mental health disorder, most commonly depression, anxiety, and substance use disorders (3). Consistent with this burden, individuals with gambling disorder have markedly elevated rates of suicidality, with lifetime suicide attempt estimates ranging from 17% to 24% (4). Financial consequences are common and often include substantial debt, legal involvement, and occupational impairment (5, 6). These harms frequently extend beyond the individual to families through financial instability and emotional distress (5, 6).

Despite the substantial burden associated with gambling disorder, the evidence base for residential treatment remains limited. Most published studies focus on outpatient psychosocial interventions, particularly cognitive behavioral therapy and motivational interviewing (7). Mapping and systematic reviews suggest modest to moderate short-term reductions in gambling behavior following these interventions, with treatment effects often diminishing beyond 6 to 12 months (8, 9). Pharmacologic trials have produced mixed results, with no consistently effective medication identified across populations (10). Many studies rely on short follow-up periods and focus on symptom reduction rather than longer-term outcomes such as sustained abstinence, functional recovery, or quality of life (11–13).

Residential treatment programs represent higher-intensity interventions for individuals with severe gambling disorder and prior treatment failures, typically providing several months of structured daily schedules, integrated psychiatric and addiction care, and restriction of access to gambling opportunities during early recovery (14). Prior studies have demonstrated benefits at discharge from short-term inpatient stabilization programs, including improvements in gambling-related attitudes and beliefs (15), and a limited number of investigations have extended follow-up to approximately one year, with 41.6% to 55% of clients remaining abstinent at one year post-discharge (16–18). However, outcomes specific to residential programs and longer-term follow-up data for these high-intensity interventions remain largely absent from the literature. This gap is particularly important given the chronic and relapsing course of gambling disorder (19, 20), as longitudinal studies indicate that relapse risk persists and may increase over time. Many individuals resume problematic gambling several years after treatment (21), often in the context of psychosocial stressors, financial pressures, and renewed exposure to gambling environments (6, 22). At the same time, recovery is increasingly understood as a multidimensional process, in which improvements in mental health, financial stability, and overall functioning are as clinically meaningful as abstinence alone (7). This complexity is reflected in many residential programs that require co-occurring substance use disorders for admission (23). Despite the intensity and resource demands of residential care, the lack of extended follow-up data limits understanding of the durability and broader impact of these interventions.

The present study evaluates long-term outcomes over an eight-year follow-up period (2017 - 2025) among individuals who attended a no-cost residential gambling treatment program in Los Angeles, California requiring co-occurring substance use disorder for admission. Former clients were contacted for structured phone interviews assessing gambling behavior, psychiatric symptoms, functional outcomes, and perceived treatment impact, addressing this gap in the residential gambling treatment literature.

## Materials and methods

### Study design and participants

The residential program is embedded within a larger 138-bed faith-based facility in Los Angeles, California, with approximately 5–10 beds designated for the gambling treatment program at any given time. The facility is funded through state support and private donations and is provided at no cost to clients. Care is delivered by an interdisciplinary team including psychiatrists, psychologists, licensed therapists, specialist addiction clinicians, spiritual counselors, peer sponsors, career advisors, and financial counselors. Psychiatric care includes weekly psychiatrist contact for medication management and treatment of co-occurring psychiatric and substance use disorders. A co-occurring substance use disorder was required for admission; a diagnosed psychiatric disorder was not. The program integrates gambling-focused interventions, substance use treatment, spiritual programming, and psychiatric care within a structured daily schedule. All participants completed a comprehensive intake assessment including standardized measures of gambling behavior, substance use, psychiatric symptoms, and functional outcomes. All former clients who attended the program over an eight-year period (2017 - 2025) were eligible for follow-up assessment and were contacted during a single outreach period.

Participants were contacted by phone and invited to complete a follow-up interview. Participation was voluntary, and verbal informed consent was obtained prior to beginning the interview. Interviews were conducted between six months to eight years post-discharge to assess long-term outcomes. The final sample consisted of 36 participants who completed the follow-up assessment out of the total 146 former clients who entered the program during this eight-year period and were contacted. Of the remaining 110 non-responders, 6 declined to complete the interview, 31 listed phone numbers were no longer in service, and 73 did not respond after six call attempts. The response rate of participants with in-service phone numbers was 33.0%, the response rate for all former clients was 24.7%.

Of participants, 27 were classified as treatment completers and 9 as treatment non-completers. Treatment completion was operationalized as a clinical determination made by the full multidisciplinary treatment team: the team formally assessed whether each participant had achieved their individualized treatment goals and required residential level of care. This determination was not defined by any other fixed metric. Treatment non-completion encompassed participants who left the program prior to a team-determined discharge decision (including those who left against clinical advice) and participants who were administratively discharged for violation of program rules.

### Follow-up assessment

Follow-up assessments were conducted by phone and lasted approximately 30 minutes to one hour. The interview consisted of a structured questionnaire combined with open-ended questions assessing the same domains measured at intake: gambling behavior, substance use, psychiatric symptoms, and functional outcomes. This allowed for direct comparison of participant functioning from intake to follow-up. The interview also included open-ended questions exploring perceived impact of treatment and contextual factors related to recovery and relapse.

### Gambling outcomes

Gambling behavior since discharge was assessed using structured self-report questions, including occurrence of gambling, time to first post-discharge episode, and contextual factors explored via open-ended prompts. Gambling disorder severity at intake was assessed using the National Opinion Research Center DSM Screen for Gambling Problems (NODS), a validated 17-item instrument derived from DSM diagnostic criteria that classifies individuals as non-problem (0), at-risk (1–2), problem (3–4), or pathological/severe (5 or more) (24).

Current gambling behavior was assessed by asking time since the most recent bet, frequency, days gambled in the past 30 days, and typical financial losses.

Gambling urges were assessed at both intake and follow-up. Participants reported the percentage of time experiencing urges over the past seven days (0–100) and craving strength (0–100). These 0–100 single-item scales were administered as part of the program’s standard clinical intake assessment and were repeated identically at follow-up to allow within-person comparison. This format is consistent with visual analog scale approaches used in addiction research to assess subjective states including craving intensity and quality of life (25). They also rated the degree to which urges interfered with normal activities and overall life satisfaction (both 0–100).

### Substance use

Substance use was assessed by asking participants to report the number of days alcohol or cigarettes were used over the past 30 days, identical to the intake assessment. Participants were also asked to describe how substance use was connected to gambling behavior before treatment and after discharge, consistent with prior work on co-occurring gambling and substance use disorders (26, 27).

### Psychiatric symptoms

Depressive symptoms were assessed using the Patient Health Questionnaire nine-item scale (PHQ-9), with scores ranging from 0 to 27. Anxiety symptoms were assessed using the Generalized Anxiety Disorder two-item scale (GAD-2), with scores ranging from 0 to 6. These standardized measures were identical to those administered at intake, allowing for within-person comparison of psychiatric symptom changes over time.

### Functional outcomes

Participants reported use of coping strategies learned during the program and identified skills most important for recovery. Engagement in post discharge supports such as therapy, mutual support groups, sponsorship, and medication management was assessed.

Functional outcomes included employment status, housing stability, and personal annual income. Participants rated their overall life satisfaction on a 0–100 scale and the degree to which gambling urges interfered with normal activities on a 0–100 scale.

### Statistical analysis

All quantitative analyses were conducted in R. Within-person changes from intake to follow-up were evaluated using paired t-tests for normally distributed measures (PHQ-9, GAD-2, craving strength, life satisfaction, interference with normal activity, percentage of time with urges). Wilcoxon signed-rank tests were used for non-normally distributed substance use variables (30-day alcohol and smoking use).

The relationship between treatment completion and gambling post discharge was examined using Fisher’s exact test, with odds ratios and 95% confidence intervals calculated.

Time to first gamble was analyzed using Kaplan-Meier survival curves. The survival analysis modeled the time from discharge to the first gambling behavior. Participants who gambled post-discharge contributed their observed number of days until the event, while those who did not gamble were right-censored at their follow-up assessment time. Additionally, a Cox proportional hazards model was used to examine treatment length of stay (in days) as a continuous predictor of time to first gambling episode, allowing for assessment of whether longer treatment duration was associated with delayed returned to gambling beyond the binary completion/non-completion distinction.

Among participants who gambled post-discharge, Wilcoxon rank-sum tests examined whether treatment completion predicted gambling severity, operationalized as (a) the number of gambling days in the past 30 days and (b) craving strength at follow-up.

### Qualitative analysis

Open-ended responses were analyzed using reflexive thematic analysis following established procedures (28, 29). Coding was primarily inductive, with themes generated from the data rather than imposed from a predetermined framework, consistent with the exploratory nature of the qualitative component. Interview responses were reviewed in full by the study team. Preliminary themes were identified independently and then discussed among authors to develop a shared coding framework. Disagreements were resolved through iterative team discussion and consensus, with no formal adjudication threshold required. Thematic saturation was not formally assessed given the fixed sample size; however, themes were considered sufficiently developed when no new conceptual content emerged across the final interviews reviewed. No qualitative analysis software was used; coding was conducted manually through systematic review and annotation of interview transcripts. Interviews were conducted by the first author (J.I.), a medical student trained on the study protocol and interview procedures who had no prior relationship with participants and was not involved in their clinical care. To enhance reflexivity, the analysis team included members with diverse backgrounds, medical students, clinical researchers, and psychiatrists with expertise in addiction treatment and gambling disorder, who brought varied perspectives to theme development and interpretation. A standardized interview guide was used across all interviews to ensure consistency.

## Results

### Quantitative analysis

#### Sample characteristics

The final sample consisted of 36 participants (75.0% male, 25.0% female) who completed follow-up assessments six months to eight years post-discharge (**Table 1**). **Table 1** also presents intake demographic and clinical characteristics for all 146 former clients who were contacted (“All Clients” column), including both responders and non-responders, to allow comparison of the responding sample against the full client population. Of responders, the mean age was 43.7 years (SD = 13.6, range: 24-68) at intake and 47.5 years (SD = 13.3, range: 27-71) at follow-up. The sample was predominantly Caucasian (72.2%). Family history was notable, with 22.2% reporting family substance abuse problems and 19.4% reporting family gambling problems. Nearly all participants (97.2%) met criteria for severe gambling disorder based on NODS scores at intake, with only 2.8% classified as moderate. The majority of participants (77.8%) had been treated for mood disorders in the 12 months prior to intake, while 8.3% had been treated for anxiety disorders. Employment status improved substantially from intake to follow-up, with employed participants increasing from 25.0% to 66.7% and unemployed participants decreasing from 75.0% to 8.3%. At follow-up, 25.0% were students, retired, disabled, or homemakers.

**Table 1:** Participant demographic and socioeconomic characteristics at intake and follow-up (N = 36).

	Responders only (N = 36)		All clients (N = 146)	
Variable	Range/N	M (SD)/%	Range/N	M (SD)/%
Demographics				
Age	24-68	43.7 (13.6)%	19-72	40.9 (12.8)%
Gender				
Male	27	75.0%	119	81.5%
Female	9	25.0%	27	18.5%
Race/ethnicity				
Caucasian	26	72.2%	112	76.7%
Non-Caucasian	10	27.8%	34	23.3%
Employment status				
Employed	9	25.0%	34	23.3%
Unemployed	27	75.0%	107	73.3%
Other	0	0.0%	5	3.4%
Personal annual income				
Under $25, 000	2	5.6%	19	13.0%
$25, 000-$49, 999	11	30.6%	40	27.4%
$50, 000-$74, 999	8	22.2%	34	23.3%
$75, 000-$99, 999	6	16.7%	26	17.8%
$100, 000 or more	9	25.0%	27	18.5%
Family history				
Family Substance Abuse Problems	8	22.2%	37	25.5%
Family Gambling Problems	7	19.4%	33	22.8%
Psychiatric diagnoses treated in past 12 months				
Mood Disorders	28	77.8%	112	77.2%
Anxiety Disorders	3	8.3%	16	11.0%
Gambling behavior				
Gambling severity based on NODS				
Severe Gambling Disorder	35	97.2%	144	99.3%
Moderate Gambling Disorder	1	2.8%	1	0.7%
Gambling losses in past 30 days				
No losses	9	25.0%	34	23.4%
Under $10, 000	13	36.1%	82	56.6%
$10, 000 or more	14	8.9%	29	20.0%
Primary gambling type				
Card games	12	33.3%	53	36.6%
Sports betting	10	27.8%	34	23.4%
Slot machines	8	22.2%	28	19.3%
Other	2	5.6%	8	5.5%
Multiple forms	4	11.1%	22	15.2%

Demographics include age, gender, race/ethnicity, employment, income, and family history. Psychiatric diagnoses represent conditions treated in the 12 months prior to intake. Gambling variables include disorder severity (NODS), past 30-day losses, and primary gambling type. Employment increased from 25.0% at intake to 66.7% at follow-up. Nearly all participants (97.2%) met criteria for severe gambling disorder at intake. The “Responders Only” column presents data for the 36 participants who completed the follow-up assessment; the “All Clients” column presents corresponding intake data for all 146 former clients who were contacted, including both responders and non-responders. Demographic and clinical characteristics were broadly similar across these two groups, supporting the representativeness of the responding sample. Psychiatric diagnoses reflect conditions for which participants received treatment in the 12 months prior to intake; categories are not mutually exclusive and do not sum to 100%. A diagnosed psychiatric disorder was not required for program admission.

#### Treatment exposure

Among all participants, median length of stay was 215.5 days (IQR: 130.8-343.8 days, range: 6–861 days), and 27 participants (75.0%) completed the full residential treatment program (**Table 2**).

**Table 2:** Treatment exposure and post-discharge gambling patterns.

Variable	Range/N	Median (IQR)/%
Treatment exposure		
Length of stay (days)	6-861	215.5 (130.8-343.8)
Post-discharge gambling behavior		
No post-discharge gambling (cumulative)	14	38.9%
6 months–2 years	4	44.4%
3–4 years	7	58.3%
5–6 years	1	12.5%
7–8 years	2	28.6%
Gambled After Discharge (cumulative)	22	61.1%
6 months–2 years	5	55.6%
3–4 years	5	41.7%
5–6 years	7	87.5%
7–8 years	5	71.4%
Slip only	2	5.6%
Returned to use, then stopped	10	27.8%
Returned to use		
Low frequency (1x/week–1x/month)	4	18.2%
High frequency (Daily–2-3x/week)	6	27.3%
Days Gambled in Last 30 Days* (cumulative)	0-30	0.0 (0.0-6.5)
6 months–2 years		4.0 (2.0–7.0)
3–4 years		1.0 (0.0–5.0)
5–6 years		0.0 (0.0–7.5)
7–8 years		0.0 (0.0–0.0)
Discharge to First Gambling Behavior (days)*	1-1825	142.5 (90.0-502.3)

Treatment exposure and gambling outcomes following discharge. Length of stay ranged from 6 to 861 days (median = 215.5). Most participants (61.1%) gambled after discharge, while 38.9% maintained complete abstinence. Among those who gambled post-discharge (n = 22), outcomes included slip only (<3 bets; 5.6%), returned to use then stopped (no gambling in past 6 months after period of regular use; 27.8%), and returned to use (gambling in past 6 months) at low (18.2%) or high frequency (27.3%). Median time to first gambling behavior was 142.5 days. At follow-up, median gambling days in the past 30 was 0.0, reflecting high rates of re-abstinence. Post-discharge gambling rates varied across follow-up intervals (55.6% at 6 months–2 years, 41.7% at 3–4 years, 87.5% at 5–6 years, and 71.4% at 7–8 years); higher rates in longer follow-up groups likely reflect greater cumulative time at risk rather than worse current functioning, as median gambling days in the past 30 days at follow-up were low across all intervals (4.0, 1.0, 0.0, and 0.0 days, respectively). *Gambling days in the past 30 days calculated among participants who reported any post-discharge gambling. Comparisons across follow-up intervals are descriptive only; subgroup sample sizes were small (n = 7-12) and formal statistical testing was not performed.

#### Within-person changes from intake to follow-up

Participants showed large and statistically significant improvements across multiple domains from intake to follow-up (**Table 3**). All gambling-related measures demonstrated substantial reductions: strength of urges to gamble decreased by 24.4 points on a 0–100 scale (p = 0.001), percentage of time experiencing urges decreased by 30.3 percentage points (p < 0.001), and gambling interference with normal activities decreased by 58.6 points (p < 0.001). Life satisfaction increased by 21.3 points (p = 0.005). Improvements in gambling-related measures were consistent across follow-up intervals, with reductions in craving strength ranging from 20.1 to 29.0 points, reductions in percentage of time experiencing urges ranging from 22.9 to 35.1 percentage points, and reductions in gambling interference ranging from 44.4 to 77.1 points across the four time-based subgroups, supporting the interpretation that these improvements are not attributable to any single follow-up window (**Table 3****).**

**Table 3:** Within-person changes in mental health and gambling-related outcomes from intake to follow-up.

	Intake		Follow-up			
Variable	Range/N	M (SD)	Range/N	M (SD)	Mean change	p-value
Mental health						
PHQ-9 score	11-11	5.6 (2.6)	0-24	6.2 (6.7)	+0.7	0.591
GAD-2 score	0-4	0.9 (0.9)	0-6	2.0 (1.0)	+1.1	0.005
Life satisfaction	0-75	45.1 (21.6)	0-100	66.2 (28.6)	+21.3	0.005
Gambling related measures						
Strength of urges to gamble (cumulative)	0-92	45.6 (26.5)	0-100	22.4 (32.3)	-24.4	0.001
6 months–2 years				22.8 (33.9)	-20.1	
3–4 years				22.5 (31.3)	-24.6	
5–6 years				23.4 (34.1)	-24.4	
7–8 years				20.3 (37.4)	-29.0	
% of time experiencing urges (cumulative)	0-85	47.3 (26.1)	0-100	17.8 (25.8)	-30.3	<0.001
6 months–2 years				20.6 (26.7)	-22.9	
3–4 years				14.0 (20.3)	-35.1	
5–6 years				20.8 (25.6)	-28.4	
7–8 years				17.6 (36.8)	-32.6	
Gambling interference with activities (cumulative)	0-100	70.0 (29.2)	0-100	13.0 (27.9)	-58.6	<0.001
6 months–2 years				14.8 (31.9)	-44.4	
3–4 years				12.0 (30.0)	-61.2	
5–6 years				16.4 (31.1)	-52.0	
7–8 years				8.3 (18.6)	-77.1	

Within-person changes in mental health and gambling-related outcomes from intake to follow-up. Depression symptoms (PHQ-9) showed no significant change (p = 0.591), while anxiety symptoms (GAD-2) increased slightly but significantly (p = 0.005). Life satisfaction improved substantially (p = 0.005). All gambling-related measures showed large and statistically significant improvements: strength of urges to gamble decreased by 24.4 points (p = 0.001), percentage of time experiencing urges decreased by 30.3 percentage points (p < 0.001), and gambling interference with normal activities decreased by 58.6 points (p < 0.001). Positive values indicate increases; negative values indicate decreases from intake to follow-up.

Depression scores, as measured by the Patient Health Questionnaire-9 (PHQ-9), showed no significant change from intake to follow-up (p = 0.591). Anxiety scores, measured by the Generalized Anxiety Disorder 2-item (GAD-2), showed a small but statistically significant increase of 1.1 points (p = 0.005).

#### Post-discharge gambling patterns and recovery trajectories

Among all participants, 22 (61.1%) reported gambling at least once after discharge, while 14 (38.9%) maintained complete abstinence throughout the follow-up period (**Table 2**). The time from discharge to first gambling episode varied widely, ranging from 1 day to 1, 825 days (approximately 5 years), with a median of 142.5 days (IQR: 90.0-502.3 days). The median time from discharge to follow-up was 3.71 years. Follow-up assessments were relatively evenly distributed across the study period, with 25.0% of participants assessed between 6 months and 2 years after discharge, 33.3% between 3 and 4 years, 22.2% between 5 and 6 years, and 19.4% between 7 and 8 years (**Table 4**). Post-discharge gambling rates and recovery trajectories were relatively consistent across follow-up intervals, ranging from 41.7% to 87.5% across subgroups, with no clear directional trend by time since discharge. Notably, participants in the longer follow-up groups (5 to 6 and 7 to 8 years) reported minimal recent gambling at the time of assessment despite higher cumulative rates of post-discharge gambling, consistent with longer time at risk rather than worse current functioning (**Table 2****).**

**Table 4:** Distribution of follow-up intervals (N = 36).

Follow-up interval	N	%
6 months–2 years	9	25.0%
3–4 years	12	33.3%
5–6 years	8	22.2%
7–8 years	7	19.4%
**Total**	**36**	**100.0%**

Median follow-up: 3.71 years (IQR: 2.03–5.74 years; range: 0.5–8.31 years). Follow-up duration defined as time from discharge to follow-up assessment.

Bold values indicate subgroup totals or primary outcome measures and are included for emphasis and ease of interpretation.

Participants who gambled post-discharge demonstrated three distinct outcome patterns. Two participants (9.1% of those who gambled; 5.6% of total sample) experienced only brief slips (fewer than three discrete episodes with rapid return to recovery and no progression to regular use). Ten participants (45.5% of those who gambled; 27.8% of total sample) returned to regular gambling but subsequently achieved sustained abstinence, having not gambled in the six months prior to follow-up. The remaining 10 participants (45.5% of those who gambled post-discharge; 27.8% of total sample) were actively gambling or had regularly gambled within the six months prior to follow-up: 6 participants engaged in high-frequency gambling (daily to 2–3 times weekly), while 4 participants gambled at low frequency (weekly to monthly).

The median number of gambling days in the 30 days prior to follow-up was 0 (IQR: 0.0–6.5), with 60.0% reporting zero gambling days, reflecting the high rate of re-abstinence among those who gambled post-discharge.

#### Treatment discharge status and gambling outcomes

Treatment discharge status was associated with post-discharge gambling patterns. Fisher’s exact test revealed that participants who left treatment prior to staff-initiated discharge had approximately 7 times higher odds of post-discharge gambling compared to those discharged by clinical staff after completing care (OR = 7.08, p = 0.062). Among the 9 participants who left treatment prior to staff-initiated discharge, 8 (88.9%) gambled during the follow-up period, compared with 14 of 27 (51.9%) among those discharged after completing treatment. Additionally, those who left prior to staff-initiated discharge experienced first gambling episodes significantly sooner than those who completed treatment (log-rank χ² = 5.5, p = 0.02; hazard ratio (HR) = 3.26), with the majority gambling within the first year after discharge.

#### Time to first gambling episode

**Figure 1:**
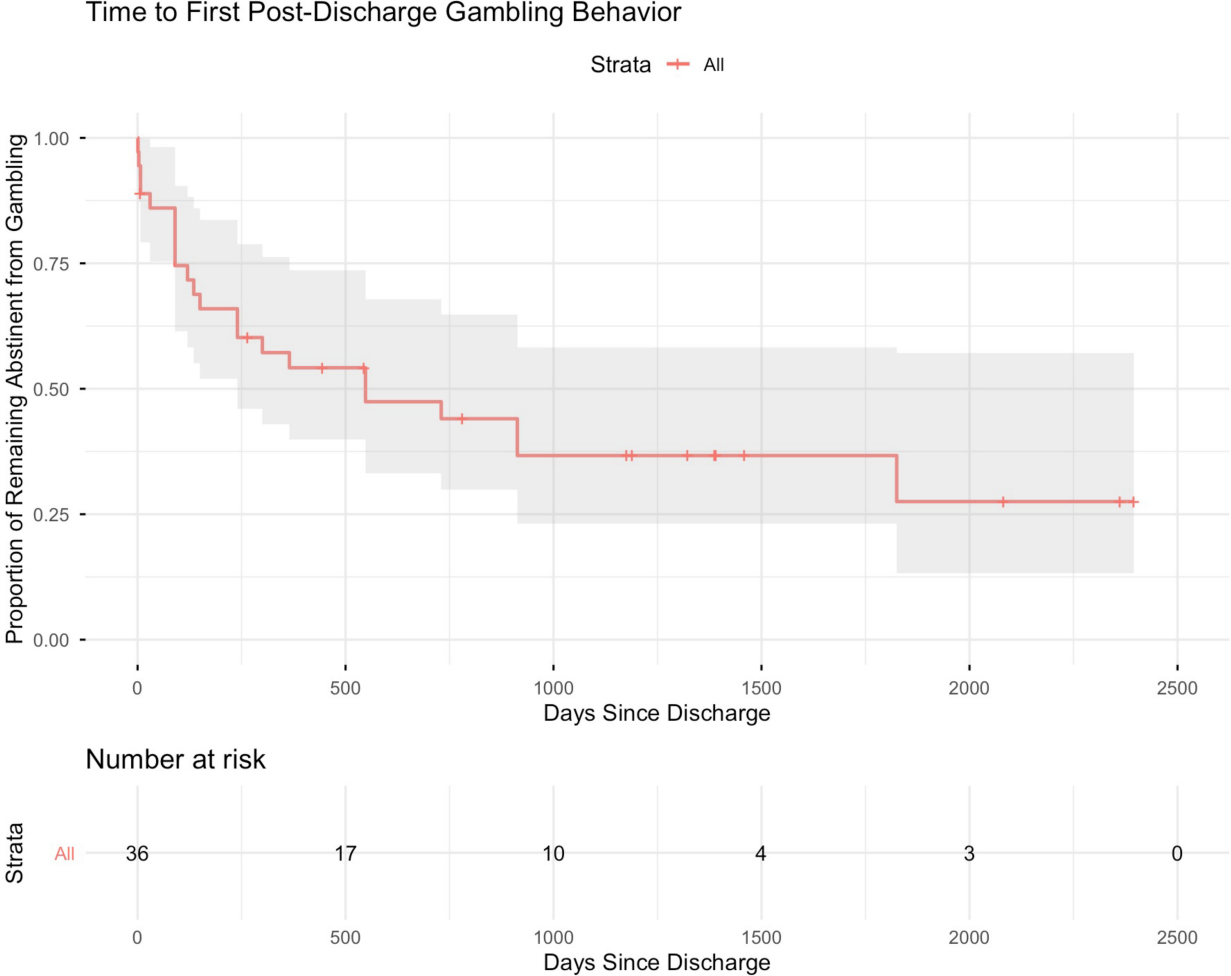
Time to first gambling behavior following discharge. Kaplan-Meier survival curve showing probability of remaining abstinent from gambling after discharge. Risk was highest in the first three months, with 25% gambling by 90 days. The median time to first gambling episode was 142.5 days (IQR: 90.0-502.3 days) for those who gambled post-discharge and 548 days (95% CI: 332–678 days) for the total number of participants. Shaded areas represent 95% confidence intervals, which widen at later time points due to decreasing numbers at risk and increasing uncertainty in survival estimates.

Kaplan-Meier survival analysis indicated that risk of first post-discharge gambling was highest within the first three months following discharge, with 25.4% of participants gambling by 90 days (**Figure 1**). The probability of remaining abstinent declined steadily over time, with an estimated median time to first gambling episode of 142.5 days for those who gambled post-discharge. Confidence intervals widened at later time points due to the decreasing number of participants remaining at risk, reflecting increased uncertainty in long-term survival estimates given the modest sample size (n = 36).

#### Treatment duration and relapse risk

A Cox proportional hazards model examining treatment length of stay as a continuous predictor indicated that longer treatment duration was associated with a lower hazard of first post-discharge gambling behavior; however, this association was not statistically significant (HR = 0.999, 95% CI [0.996, 1.002], p = .40). Although the per-day effect was small, scaling the estimate suggests that an additional 90 days of treatment corresponded to approximately a 10% reduction in hazard, though this effect did not reach statistical significance in the sample.

#### Gambling severity among those who relapsed

Among the 22 participants who gambled post-discharge, treatment discharge status did not predict gambling severity. There were no statistically significant differences between those who completed treatment and those who left prior to staff-initiated discharge in the number of gambling days in the past 30 days (p = 0.2) or craving strength at follow-up (p = 0.65). This indicates that while treatment discharge status predicts whether and when post-discharge gambling occurs, it does not predict the intensity or severity of gambling behavior among those who gambled.

### Qualitative themes

#### Environmental restructuring disrupted gambling behavior patterns

Participants described the residential environment as systematically dismantling the contextual conditions that enabled gambling behavior. Gambling was characterized as flourishing within specific enabling factors including privacy, unstructured time, digital access, and financial autonomy. By removing these variables, the residential program appeared to interrupt the automaticity of gambling and redirected cognitive resources toward self-reflection and skill development.

Constant visibility and peer accountability emerged as primary mechanisms of behavioral change. The secrecy inherent to gambling was replaced by a high-visibility social structure in which ongoing proximity to staff and peers created barriers to the emotional withdrawal that typically precedes gambling episodes. The removal of money and devices functioned as external controls that eliminated the need for constant self-negotiation. Participants reported that outside the residential setting, they expended considerable mental energy resisting or justifying gambling-related decisions. The residential environment allowed urges to be experienced without the possibility of action, creating what participants described as a protective “bubble” in which new skills could be practiced while consequences were managed by the program. As one participant described: [The program] gave me a bubble to grow in that was separated from all of the other chaos in my life at the time. I needed that inpatient experience. No outpatient experience could have helped me that way.’

#### Development of urge management skills through structured practice

Participants described a fundamental shift in how they responded to gambling urges, moving from an impulse-action sequence toward a reflective process of observation. This shift was achieved through internalization of “the pause, ” a cognitive buffer between urge and behavior developed through repetitive practice in a controlled environment. Many participants used the term “urge surfing” to describe learning to tolerate the discomfort of cravings without seeking immediate relief through gambling. One participant explained, “I’m able to keep my initial reactions private now. I don’t want to react so quickly. I want to process [the urge], that’s the key component.” Another participant similarly noted: “They allow me to tolerate the discomfort without taking action. I’m certain I wouldn’t have had the same level of practice and mastery over these skills if I wasn’t [in] the safe environment of [the program] and through its extensive counseling.”

Accountability to others remained important even after discharge, with relationships to sponsors or peers helping to interrupt the secrecy and rumination associated with relapse risk. Participants who gambled post-discharge often described gambling reentering their lives through small exceptions rather than overwhelming urges. In response, those maintaining abstinence adopted firm rules to reduce ambiguity, avoiding even ambiguous gambling-related activities. Participants also described an identity transformation extending beyond behavioral change, with many entering treatment viewing themselves primarily through the lens of financial losses but leaving with a reframed sense of self-worth.

#### Sequential recovery processes requiring extended time

Treatment duration emerged as a critical variable, with participants identifying a chronological sequence beginning with initial stabilization, progressing to skill acquisition, and culminating in identity integration. Early treatment was often characterized by acute withdrawal symptoms and co-occurring psychiatric distress, suggesting that intensive psychotherapy may be ineffective during acute crisis states. Flexible length of stay allowed recovery to proceed at an individualized pace, which was particularly important for participants with trauma histories or unstable life circumstances.

Extended time in treatment allowed participants to experience urges, interpersonal conflict, and emotional setbacks while still within the safety of the program structure. Participants who left treatment prematurely often felt unprepared for community reentry and reported possessing knowledge about recovery strategies but lacking the automaticity necessary to deploy these skills under stress. One participant contrasted residential treatment with outpatient care: “It’s not like going to [gamblers anonymous], setting some goals, then going home and not following through on them. The routines that [the program] makes you build … are the same routines you try to keep once you leave.”

#### Structured routine as a substitute for gambling-organized time

Many participants described gambling as having served as the primary organizing activity in their lives, providing stimulation and a sense of urgency. The residential program substituted as a daily organizer and externally imposed routines. Structured activities including fitness and group participation filled the day and weakened associations between unstructured time and gambling. Participants identified boredom and unstructured time as high-risk states that preceded gambling urges.

Post-discharge outcomes often depended on whether participants could recreate structured purpose in their daily lives. Those who transitioned into employment or maintained scheduled recovery activities reported greater stability, while participants who returned to unstructured environments identified boredom as a primary trigger for returning to use.

#### Geographic and emotional buffering from high-risk relationships

Gambling behavior was described as highly context-dependent, often triggered by specific relational stressors. For many participants, home environments were characterized by active addiction or interpersonal conflict, and residential treatment provided geographic and emotional distance from these triggers. Premature re-exposure to high-risk environments often overwhelmed newly acquired coping skills, with participants who returned home to family members with active gambling disorder describing immediate return to gambling.

The residential period allowed participants to build emotional capacity and reassess relationships with clinical support before returning home. Gambling following early departure was often attributed to immediate re-exposure to the stressors that had precipitated gambling, rather than to a lack of motivation or skill deficits. As one participant described: “The program helped me learn enough about myself and the toxic environments that I was in when I was gambling. It made me open my eyes to how much emotional pain I was experiencing at home from my husband and how that might be connected to my addiction. I was able to set personal boundaries that helped me prioritize my own well being.”

#### Maintenance of structure and support post-discharge

The transition from residential to community living emerged as a period of extreme vulnerability, with post-discharge outcomes depending heavily on whether structural elements of the program could be maintained. Practical preparation during residential treatment shaped long-term outcomes, with employment planning and financial management identified as essential. Alumni contact and ongoing follow-up helped preserve the sense of belonging developed during treatment. One participant described the importance of establishing post-discharge community: “I also knew I needed to find community on the outside … finding people who were going to have long-term sobriety on the outside. [The program] gave me those connections and the time to find them which made my transition to the outside world much more sustainable and supported.”

Successful transitions were characterized by viewing recovery as a lifelong commitment and maintaining engagement with mutual support groups. Participants emphasized that maintaining the internal structure of the program after external structure was removed was critical for sustained recovery.

#### Reconceptualization of financial management

The enforced period without financial access during residential treatment appeared to facilitate a shift in how participants related to money, with money reconceptualized as a regulated risk factor. Participants recognized that financial stability could be as dangerous as financial crisis and implemented long-term financial guardrails including third-party oversight or conservatorships. During treatment, experiencing urges without financial access demonstrated that cravings would eventually subside without action, representing a fundamental shift from viewing urges as requiring immediate response. As one participant reflected: “[The program] empowered me … changing my mindset about responding instead of reacting, learning how to control my instant emotions, being more honest with what my actions will lead to if I don’t stop now.”

Participants also identified “gray area” financial behaviors such as cryptocurrency trading or high-risk stock options as psychologically equivalent to gambling. Poor outcomes were often associated with inadequate financial preparation at discharge, while successful long-term recovery required permanent changes in financial management.

## Discussion

This study evaluated long-term outcomes among individuals who attended a residential gambling treatment program requiring co-occurring substance use disorder for admission. Follow-up assessments conducted six months to eight years post-discharge revealed sustained improvements in gambling-related distress and functional outcomes, with heterogeneous recovery trajectories challenging binary conceptions of treatment success. While 61% of participants gambled at least once post-discharge, outcomes varied substantially: some experienced only brief slips, others returned to use then achieved sustained re-abstinence, and still others continued active use. Integration of quantitative and qualitative data identified multiple mechanisms through which residential treatment may produce durable effects: environmental restructuring that disrupts gambling behavior patterns, development of urge management skills through structured practice, sequential recovery processes requiring extended time, structured routines replacing gambling-organized time, geographic and emotional buffering from high-risk relationships, successful transition planning, and reconceptualization of financial management.

The sample represented a severely affected population with substantial psychiatric comorbidity and familial risk. Nearly all participants (97.2%) met criteria for severe gambling disorder at intake based on NODS scores, reflecting the high clinical threshold required for residential admission. The prevalence of family substance abuse problems (22.2%) and family gambling problems (19.4%) suggests exposure to adverse social dynamics in youth and early life that may have contributed to the development of gambling disorder. The high rate of mood disorder treatment (77.8% in the 12 months prior to intake) underscores the psychiatric complexity of this population, consistent with literature documenting elevated rates of depression and anxiety among individuals with gambling disorder (3).

### Durability of treatment effects beyond abstinence

The finding that participants demonstrated large and statistically significant improvements in craving strength, life satisfaction, interference with normal activities, percentage of time experiencing urges, and alcohol use from intake to follow-up is consistent with, though not causally attributable to, residential treatment, given the observational and uncontrolled nature of this study. Despite 61% of participants (22 of 36) reporting at least one gambling episode after discharge, the cohort showed substantial reductions in gambling-related distress and improved functioning. The 58.63-point reduction in interference with normal activities (p < 0.001) is particularly noteworthy, indicating that even individuals who returned to some gambling behavior experienced substantial functional recovery.

Among the 22 participants who reported any gambling after discharge, 54.5% had achieved sustained abstinence for more than 6 months at the time of follow-up, and 60% reported zero gambling days in the 30 days prior to assessment. This pattern of post-discharge gambling followed by return to abstinence suggests that residential treatment may equip individuals with skills to interrupt gambling episodes and re-establish recovery, even after initial setbacks. The median time to first gambling episode of 142.5 days among participants that gambled post-discharge indicates that most participants maintained initial abstinence for several months post-discharge, providing a foundation for applying learned skills in community settings.

These findings align with recovery frameworks emphasizing restoration of health, wellness, and quality of life over binary abstinence outcomes (30). The qualitative data support this interpretation, with participants describing fundamental shifts in their relationship to money, development of sustainable coping strategies, and integration of recovery-oriented identity even after gambling episodes. This suggests that residential treatment may confer benefits that persist independently of complete abstinence, an important consideration given the chronic and relapsing nature of gambling disorder (30).

Employment outcomes provide additional evidence of functional recovery. The increase from 25% employed at intake to 66.7% at follow-up represents substantial improvement in occupational functioning, which is a key indicator of recovery (16). This occurred despite the majority of participants experiencing at least one episode of gambling post-discharge, suggesting that improved functioning extends beyond gambling abstinence alone. The relative stability in mean annual income (from $71, 805.56 to $72, 696.43) may reflect that many participants were stabilizing employment rather than advancing careers, or that some remained in recovery-oriented activities rather than pursuing higher-paying positions.

Given that 77.8% of participants had been treated for mood disorders and 8.3% for anxiety disorders in the 12 months prior to intake, the high baseline psychiatric burden in this sample is notable. The lack of improvement in depression scores (PHQ-9) and the small but significant increase in anxiety (GAD-2) warrant careful interpretation. The residential program may have successfully addressed gambling-specific distress but been less effective at treating underlying mood and anxiety disorders requiring longer-term psychiatric intervention. Alternatively, participants may have experienced increased anxiety related to post-discharge life stressors, financial obligations, or awareness of relapse risk. The persistence of psychiatric symptoms despite improvements in gambling-related functioning underscores the importance of addressing co-occurring mental health conditions as distinct treatment targets (31).

### Heterogeneous recovery trajectories following residential treatment

The finding that participants who gambled post-discharge demonstrated three distinct outcome patterns (slips only, return to use followed by re-abstinence, and ongoing use) challenges binary conceptions of treatment success and failure. While 61% of participants gambled at least once following discharge, this single statistic obscures substantial heterogeneity in recovery trajectories. Among those who gambled, only 45% were actively gambling or had gambled within six months of follow-up, while the remainder had either experienced only brief slips (5.6%) or had returned to gambling but subsequently achieved sustained re-abstinence of six months or longer (45.5%). This suggests that residential treatment may equip individuals with skills that facilitate recovery from lapses rather than only preventing initial gambling episodes.

The capacity for re-establishing abstinence after post-discharge gambling behavior represents an important treatment outcome that is frequently overlooked in binary outcome frameworks. Ten participants (27.8% of the total sample) demonstrated that returning to regular gambling use does not preclude subsequent sustained abstinence. This pattern aligns with addiction recovery models emphasizing relapse as a common feature of the recovery process rather than treatment failure (32).

The heterogeneity of post-discharge trajectories has important implications for outcome measurement in gambling treatment research. Studies relying solely on point-prevalence abstinence rates or one year follow-up data may misclassify individuals as treatment failures when they have achieved sustained re-abstinence after an initial lapse. Stratification of outcomes by follow-up interval further supports this interpretation: participants assessed at longer intervals showed higher cumulative rates of post-discharge gambling, as expected given greater time at risk, but reported comparable or lower current gambling at the time of assessment, suggesting that longer elapsed time since discharge does not correspond to worse recovery status at follow-up. Similarly, time-to-first-gambling analyses provide important information about relapse risk but do not capture whether individuals recover from lapses. The current findings suggest that comprehensive outcome assessment should include both abstinence rates and patterns of re-abstinence among those who gamble, as well as gambling frequency and severity among those with ongoing use.

### Treatment discharge status and duration

Treatment non-completers showed substantially elevated risk for post-discharge gambling (88.9% vs. 51.9% among completers; OR = 7.08, p = 0.062) and experienced first gambling episodes significantly sooner (HR = 3.26, p = 0.02). However, discharge status did not predict gambling severity among those who gambled, with no significant differences in gambling frequency or craving intensity at follow-up between the two groups. This pattern suggests that whether a patient reached the clinical team’s treatment completion threshold predicts whether and when post-discharge gambling occurs, but not gambling intensity. The association between non-completion and poorer outcomes likely reflects multiple interacting factors. Individuals who do not reach the completion threshold may differ systematically in motivation, illness severity, social support, or external stressors. Importantly, because completion status was determined naturalistically by the clinical team rather than experimentally assigned, this variable likely captures both the clinical meaning of having achieved treatment goals and the individual-level characteristics that predicted whether goals could be reached.

Treatment duration as a continuous variable showed a non-significant trend toward reduced return to gambling risk (HR = 0.999 per day, p = .40), with each additional 90 days corresponding to approximately a 10% reduction in hazard. The lack of statistical significance likely reflects the modest sample size and wide variability in length of stay (range: 6–861 days). Notably, the continuous duration effect was weaker than the binary completion effect (OR = 7.08), suggesting that whether a patient reached the clinical team’s completion threshold may matter more than raw time in treatment.

Participants consistently described treatment as requiring sufficient time for a multi-stage process: initial stabilization from acute withdrawal and psychiatric crisis, followed by skill acquisition through repetitive practice, and culminating in identity integration. Those who left treatment prematurely often reported feeling unprepared for community reentry, possessing knowledge about recovery strategies but lacking the automaticity necessary to deploy these skills under stress. As one participant noted, residential treatment builds routines “that are the same routines you try to keep once you leave, ” suggesting that extended time allows for habituation of recovery behaviors that persist post-discharge.

### Mechanisms of residential treatment efficacy

The qualitative analysis identified several mechanisms through which residential treatment may exert its effects. Environmental restructuring dismantled the conditions enabling gambling by removing privacy, unstructured time, digital access, and financial autonomy, interrupting its automaticity. Constant visibility and peer accountability replaced the secrecy inherent to gambling, allowing urges to be experienced without the possibility of action.

The residential setting facilitated development of urge management skills through structured, repetitive practice, with skill automaticity requiring extended time that early leavers did not achieve.

Time in residence allowed for sequential recovery processes requiring acute stabilization before skill-building and identity integration can begin. This challenges assumptions that shorter treatment episodes can substitute through increased intensity, as certain recovery processes are fundamentally time-dependent (33).

The residential program also functioned as a substitute organizer for lives previously structured around gambling. Structured activities filled time and weakened associations between boredom and gambling urges. Those returning to unstructured environments consistently identified boredom as a primary relapse trigger, suggesting that the schedule may be as important as specific therapeutic interventions.

Geographic and emotional buffering from high-risk relationships emerged as another critical mechanism. Premature re-exposure to environments characterized by active addiction or interpersonal conflict often overwhelmed newly acquired coping skills.

Finally, the enforced period without financial access demonstrated that cravings would eventually subside without action, facilitating reconceptualization of money as a regulated risk factor. Many participants implemented long-term financial guardrails including third-party oversight.

Integration of quantitative and qualitative findings reveals a coherent pattern: the domains showing the largest quantitative improvements, namely gambling-related interference, craving strength, and urge frequency, correspond directly to the mechanisms participants described qualitatively, including development of urge management skills, environmental restructuring, and substitution of structured routine for gambling-organized time. Similarly, the quantitative finding that treatment non-completion predicted earlier return to gambling aligns with qualitative accounts of participants who left prematurely feeling unprepared for community reentry and lacking the skill automaticity developed through extended residential treatment.

### The critical transition period

The transition from residential to community living emerged as a period of extreme vulnerability. The median time to first gambling episode of 142.5 days (approximately 5 months) suggests a critical window during which many participants struggled to maintain abstinence in community settings. However, the wide range of time to first gamble (1 to 1, 825 days) indicates substantial individual variability, with some participants relapsing immediately upon discharge while others maintained abstinence for years.

These findings suggest that discharge planning and aftercare may be as critical as the residential treatment itself. The concept of “recovery capital, ” which is the internal and external resources that support sustained recovery, has been increasingly emphasized in substance use treatment programs (34). Programs should explicitly prepare participants for transition by helping them establish post-discharge supports, develop structured daily schedules, implement financial management systems, and create relapse prevention plans addressing specific high-risk situations (35). Provision of alumni support, such as periodic check-ins or access to the residential community, may help sustain the sense of belonging and accountability that participants identified as protective.

## Limitations

Several limitations should be considered. First, the study achieved a response rate of 24.7% overall (36 of 146 former clients), with 33.0% of those with active phone numbers completing the follow-up interview. The resulting sample size (N = 36) limits statistical power, precludes more complex analyses examining multiple predictors simultaneously, and raises the possibility of response bias, as those who participated may differ systematically from non-respondents in recovery status or willingness to disclose gambling behavior. The width of confidence intervals in the survival analysis reflects this limitation. Importantly, however, a comparison of demographic and clinical characteristics between the responding sample and all 146 former clients who were contacted revealed no substantively meaningful differences across key variables including age, gender, race/ethnicity, employment status, income, family history, psychiatric diagnoses, and gambling disorder severity (**Table 1**). This demographic comparability provides some empirical support for the representativeness of the responding sample and suggests that the findings may generalize to the broader treatment cohort, even though the possibility of unmeasured differences in recovery status between responders and non-responders cannot be fully ruled out. Additionally, given that follow-up assessments occurred up to eight years post-discharge, participants’ retrospective accounts of gambling behavior, treatment experiences, and recovery trajectories may be subject to recall bias, including memory distortion, telescoping, or selective recall, particularly for events further from the time of interview.

Second, the low overall response rate (24.7%; 36 of 146 former clients) raises important concerns regarding several forms of bias. Survivorship bias may have affected results if participants who were alive and reachable at follow-up differed systematically from those who were not. Self-selection bias is a particular concern, as individuals with more positive recovery outcomes may have been more willing to participate, potentially leading to overestimation of treatment effectiveness. Attrition bias is also possible, as the 73 individuals who did not respond after six contact attempts and the 31 with disconnected phone numbers may differ meaningfully from responders in ways that could not be assessed. Although comparison of demographic and clinical characteristics between responders and all 146 former clients revealed no substantive differences across measured variables, this comparison cannot rule out unmeasured differences in recovery status or treatment engagement. The finding of broad improvements should therefore be interpreted cautiously, as the sample may not be representative of all individuals who attended the program. The study relied on self-reported outcomes without biological verification or collateral informants. Social desirability bias may also have led some participants to underreport gambling behavior (36), though the 61% post-discharge gambling rate and willingness of participants to report recent gambling activity suggests generally candid disclosure. The 0–100 single-item scales used to assess craving strength, urge frequency, interference with activities, and life satisfaction have not been independently psychometrically validated within a gambling disorder population, which should be considered when interpreting within-person change scores.

Third, the follow-up period varied substantially across participants (range: 6 months to 8 years; median: 3.71 years, IQR: 2.03–5.74 years), introducing meaningful heterogeneity in relapse opportunity, recovery stage, recall accuracy, and exposure to post-treatment supports. To address this, outcomes were stratified by follow-up interval (**Table 2**), and improvements in gambling-related measures were consistent across subgroups, suggesting findings are not systematically driven by follow-up duration. Stratification by follow-up duration is descriptive only given small subgroup sizes (n = 7-12), and formal statistical comparisons across intervals were not performed. Participants assessed at 6 months post-discharge and those assessed at 8 years are at fundamentally different stages of recovery, and analyzing them together may complicate interpretation of outcomes. Stratification of results by follow-up duration would be informative but was precluded by the modest sample size. Future studies should incorporate multiple standardized assessment points to better characterize recovery trajectories over time.

Finally, the program required co-occurring substance use disorder for admission, limiting generalizability to individuals with gambling disorder alone. The mechanisms and outcomes identified may not fully apply to less complex presentations.

## Future directions

Future research should address these limitations through larger longitudinal studies with adequate power and multiple assessment points to examine recovery trajectories and identify vulnerability periods (37). Studies should include objective outcome measures and collateral reports to triangulate self-report.

Research is needed to identify which residential treatment components are essential versus expendable. Component analysis or dismantling studies could help streamline treatment without sacrificing effectiveness (38).

Investigation of optimal treatment duration is particularly important given residential care costs. Ideal length of stay may vary by individual characteristics, and adaptive treatment designs could test whether a minimum effective threshold exists (37).

Finally, future research should prioritize the critical transition from residential to community living. Stepped-down care including sober living, intensive outpatient programs, or mobile health technologies may bridge this gap (39–41). The finding that over half of relapsed participants achieved sustained re-abstinence suggests that interventions supporting recovery from lapses may be as important as preventing initial relapse.

## Conclusions

This study provides preliminary observational evidence that residential treatment for gambling disorder with co-occurring substance use disorder is associated with improvements in gambling-related distress and life functioning across heterogeneous recovery trajectories, though causal conclusions cannot be drawn from this uncontrolled design. While 61% of participants gambled at least once post-discharge, outcomes varied substantially: some experienced only brief slips, others returned to use then achieved sustained re-abstinence, and still others continued active use. Among those who gambled, over half (54.5%) achieved sustained re-abstinence of six months or longer, suggesting that residential treatment may equip individuals with skills to interrupt gambling episodes and re-establish recovery. Employment improved from 25% at intake to 66.7% at follow-up, indicating substantial functional gains extending beyond gambling behavior alone.

Qualitative analysis identified multiple mechanisms through which residential treatment may operate, including environmental restructuring, skill development through repetitive practice, sequential recovery processes requiring extended time, structured routines replacing gambling organization, buffering from high-risk relationships, and transformation of financial management. The findings suggest that residential treatment should be considered for individuals with severe gambling disorder, multiple prior treatment failures, or significant psychiatric comorbidity. However, programs must attend carefully to discharge planning and aftercare support, particularly during the critical first six months post-discharge when gambling risk appears highest. By integrating quantitative outcome data with detailed qualitative accounts of recovery experiences, this study contributes to understanding how intensive treatment may support long-term recovery from gambling disorder.

## Data Availability

The raw data supporting the conclusions of this article will be made available by the authors, without undue reservation.
